# Acute Epiphysitis

**Published:** 1910-01-01

**Authors:** 


### Surgery.

### ACUTE EPIPHYSITIS.

It is a curious fact, and one which is difficult to explain, that, in hospital practice at any rate, acute disease of long bones is less common than it used to be. The introduction of antiseptics can hardly be held responsible for this, as it can be for most of the changes which have altered the aspect of surgery in the last twenty years. Perhaps the most feasible explanation is that the condition is nowadays recognised in an earlier stage, and subsides in response to efficient treatment before the lesion becomes advanced. How rarely does one now see a genuine case of acute epiphysitis, for instance. In practice this is not a common disorder, and is most often associated with acute inflammatory changes in other parts of the bone. In fact, in any acute bone lesion it is almost impossible to be able to say dogmatically that it started in the epiphysis or in the medulla or under the periosteum, since after a short time all the constituents of the bone become progressively involved.

These several conditions are the result of what may be called a hæmogenous infection in the great majority of cases. Occasionally, of course, an acute periostitis and osteitis may supervene upon the infliction of a septic wound involving the periosteum. Such a condition is found now and then in the tibia following an injury over its subcutaneous surface. But it is the exception. Usually some slight injury is the determining factor; this devitalises the tissues of the affected part and lowers their resistance against invasion by any organisms which may happen to be in the blood stream; and it must be supposed that any normal individual has in his blood pathogenic micro-organisms which give rise to no symptoms as long as he continues in good health. It is probable that most people, for instance, carry some tubercle bacilli in this way.

What happens, then, as the result of an injury to the bone? The micro-organisms become arrested in the fine capillary vessels; and since in the case of a bone, the largest anastomosis of capillaries, that between the tributaries of the periosteal supply and those from the nutrient artery, takes place at the diaphyseal side of the epiphyseal line, it is here that we most often find symptoms of their activity. The organisms, generally staphylococci or streptococci accumulate, and produce first thrombosis of the small vessels in the neighbourhood, and necrosis of the cancellous tissues, partly because their blood supply is cut off and partly on account of the virulence of the toxins. The necrosis thus begun tends to spread, generally along the diaphysis, but occasionally it travels towards the epiphysis and is localised to the end of the bone. This, when it occurs, is the condition known as "acute epiphysitis."

The ultimate result varies in any given case. Usually the epiphysis becomes disintegrated and separated from the shaft along the epiphyseal line; and it is along this line that the abscess tends to track. The joint may become infected in one of two ways. The abscess may make its way into the synovial membrane by eroding the articular surface, or in those cases in which the epiphyseal line lies inside the joint, as in the upper end of the femur or of the ulna, the abscess may infect the synovial membrane by travelling along this line without necessarily destroying any part of the articular surface. Or, again, when the abscess gets to the edge of the epiphyseal line it may spread down between the periosteum and the shaft, giving rise to acute suppurative periostitis and panostitis.

If the abscess bursts into the joint cavity, an acute suppurative arthritis will, of course, be set up. But before this occurs there will often be evidence of an accumulation of fluid in the adjacent joint; and if this be withdrawn with an aspirating syringe and examined bacteriologically it will be found to be turbid, but not purulent, and to contain staphylococci or streptococci, as the case may be.

From what has been said it will be seen that, clinically, these cases are not easy to diagnose with certainty. All one has to go on is the history of a slight injury some days previously, followed by great pain and tenderness over an epiphyseal line, with possibly some effusion into the adjacent joint. In most cases the child looks flushed and profoundly ill, the temperature is persistently high, and the pulse is full, rapid and bounding.

The most important factor in the treatment is to give free exit to the pus if an abscess has formed. Since this is deep-seated, it is usually necessary in the early stages to trephine or gouge an opening in the bone; the epiphyseal line is often damaged in this procedure, but such damage may be unavoidable, and it must be remembered that if free drainage is not established the whole epiphysis will certainly perish, and further, the patient may lose his life. If an operation is performed when the epiphysis is already separated by the disease, there will be no difficulty in getting at the root of the trouble.

But the operative interference is only part, nay, a small part, of the treatment. Every means must be used to increase the resistance of the patient against the infection. The best results are obtained by giving large doses of a polyvalent anti-streptococcic serum. If the particular micro-organism can be isolated, a vaccine should be prepared at once and its administration started at the earliest possible moment. Drugs are not of great value, but quinine is the best, and it seems to be particularly efficacious if given in effervescing solution. It has two valuable properties: it is a bactericide and an antipyretic.

Great stress is laid on the importance of general treatment to increase the patient's resistance, because the most fatal variety of this disease is that in which the local lesion is apparently small, yet the patient passes rapidly into a state of profound toxæmia and dies of a general septic absorption rather than from the direct effects of the necrosis.

